# Nymphs of the common bed bug (*Cimex lectularius*) produce anti-aphrodisiac defence against conspecific males

**DOI:** 10.1186/1741-7007-8-121

**Published:** 2010-09-09

**Authors:** Vincent Harraca, Camilla Ryne, Rickard Ignell

**Affiliations:** 1 Division of Chemical Ecology, Department of Ecology, Ecology Building, Lund University, SE-223 62 Lund, Sweden; 2 Division of Chemical Ecology, Department of Plant Protection Biology, Swedish University of Agricultural Sciences, Box 102, SE-230 53 Alnarp, Sweden

## Abstract

**Background:**

Abdominal wounding by traumatic insemination and the lack of a long distance attraction pheromone set the scene for unusual sexual signalling systems. Male bed bugs (*Cimex lectularius*) mount any large, newly fed individual in an attempt to mate. Last instar nymphs overlap in size with mature females, which make them a potential target for interested males. However, nymphs lack the female's specific mating adaptations and may be severely injured by the abdominal wounding. We, therefore, hypothesized that nymphs emit chemical deterrents that act as an honest status signal, which prevents nymph sexual harassment and indirectly reduces energy costs for males.

**Results:**

Behavioural mating assays showed that males mount nymphs significantly shorter time compared to females, although initial mounting preference was the same. In support of our hypothesis, nymphs experienced the same percentage of mating with sperm transfer as females if they were unable to emit (*E*)-2-hexenal, (*E*)-2-octenal 4-oxo-(*E*)-2-hexenal and 4-oxo-(*E*)-2-octenal, from their dorsal abdominal glands. We report that the aldehydes and 4-oxo-(*E*)-2-hexenal are detected by olfactory receptor neurons housed in smooth and grooved peg sensilla, respectively, on the adult antennae, at biologically relevant concentrations. Behavioural experiments showed that application of 4-oxo-(*E*)-2-hexenal or the two aldehydes at a nymph-emitted ratio, to a male/female pair during mounting initiation, decreased mating frequency to a rate comparable to that of a male/nymph pair.

**Conclusions:**

By combining behavioural and sensory studies, we show that the nymph-specific alarm pheromone plays an important role in intra-specific communication in the common bed bug. Alarm pheromones are commonly looked upon as a system in predator/prey communication, but here we show that alarm pheromones may be used as multipurpose signals such as decreasing the risk of nymphal mating by males.

See commentary: http://www.biomedcentral.com/1741-7007/8/117

## Background

The chemical communication, as well as the mating behaviour, of bed bugs (*Cimex lectularius*) has received increased attention in recent years as bed bug infestations have increased worldwide. The occurrence of alarm pheromones has been known since the 1970 s [1] but, in the light of traumatic insemination (the only mode of copulation in the Cimicidae), new discoveries concerning the usage of alarm pheromones by bed bugs are changing the common view that alarm pheromones are used as a predator defence system only [2].

All developmental stages and both sexes of *C. lectularius *are obligate blood-feeders and they form large aggregations within close proximity of their human host. Bed bugs remain hidden inside their refuge where they mate, moult and oviposit until their next nocturnal blood meal on the resting host [3,4]. Mating is exclusively traumatic, with the male piercing a secondary genital opening in the female with its lanceolate paramere and ejaculating directly into the abdominal cavity [3,4]. Mating occurs throughout the adults' life and hence polygamous mating is common [3-5]. Multiple mating injuries have been shown to be costly for females, probably due to increased immunological threat [6], and lead to a reduced life span [7].

Mating is closely associated with blood feeding and male attention is directed to any large, newly blood-fed bed bugs [8,9]. Hence, males display characteristic mounting behaviour when presented to an engorged individual regardless of sex [5,10]. If the mounted mate is a male or a large nymph, the males respond by a quick dismount and mating does not occur [5]. This suggests a lack of long-distance sex identification signals and that identification occurs after mounting [10].

It is well established that stressed bed bugs of all stages and sexes release an alarm pheromone, causing the dispersal of conspecifics [4,11]. However, the alarm pheromone has a dual function in bed bugs, where a mounted male responds by releasing alarm pheromone to prevent homosexual mating [10], in addition to adopting specific behaviours [5]. In contrast, females do not release alarm pheromone when mounted by a male [10,11]. The alarm pheromone is produced by glands located between the first and second pairs of legs on the methatorax of adults and dorsally on the nymph abdomen [4]. The alarm pheromone is composed of (*E*)-2-hexenal and (*E*)-2-octenal [12,13], both of which are detected by olfactory receptor neurons (ORNs) housed in smooth peg sensilla on the adult antenna [14]. Female and male bed bugs produce different ratios of the two aldehydes, with both sexes releasing a larger amount of (*E*)-2-hexenal than nymphs [12]. In addition to the aldehydes, last nymphal stages also produce two oxygenated aldehydes, 4-oxo-(*E*)-2-hexenal and 4-oxo-(*E*)-2-octenal [[15], Liedtke *et al*., unpublished data]. The behavioural role of these nymph-specific compounds, or the significance of the reversed ratio of the two aldehydes, has not yet been established.

We hypothesize that the nymph-emitted compounds are used by nymphs as an identification signal, which deters mate-seeking males. After establishing that male bed bugs are able to detect these compounds, using single sensillum recordings, we predict that by removing the possibility for nymphs to emit their pheromone, nymphs would experience a higher rate of mating than non-manipulated nymphs. In addition, the addition of the major nymph-specific compound 4-oxo-(*E*)-2-hexenal, as well as a reversed ratio of the two aldehydes would result in mating disruption of adult bed bugs.

## Results

### Male mounting interest to nymphs

Males did not distinguish between nymphs and females of similar size as they mounted females (*n *= 30) and nymphs (*n *= 29) equally often, with a mean frequency of 0.61 and 0.48, respectively (Kruskal Wallis: χ^2 ^= 1.089, *P *= 0.297). In contrast, males spent significantly more time in mounting position on females (964 ± 645 ms) than on nymphs (142 ± 427 ms; ANOVA: F = 32.787, *P *< 0.001). Mounting of females resulted in 79.2% mating with sperm transfer, whereas no mating with sperm transfer was observed in mounted nymphs (Mann Whitney: *Z *= -4.574, *P *< 0.001).

### Removal of nymph alarm pheromone

**Figure 1:**
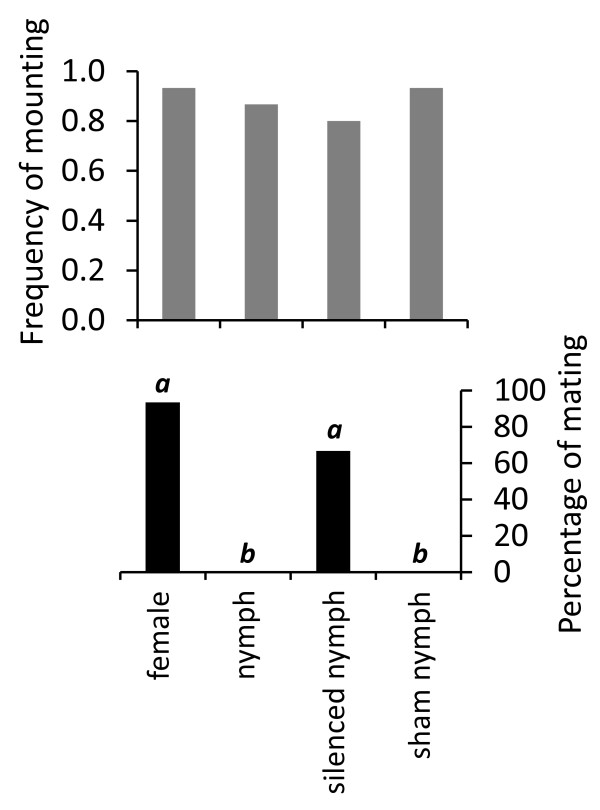
**Mating repulsion of male *Cimex lectularius *is regulated by the nymph-emitted alarm pheromone**. (a) Frequency of male mounting on females and nymphs (*n *= 15 for each treatment) is the same (Kruskal-Wallis test: χ^2 ^= 1.749, *P *= 0.626). (b) The percentage of mating with females (*n *= 14), nymphs (*n *= 12), silenced nymphs (with covered dorsal abdominal glands (*n *= 13) and sham-treated nymphs (*n *= 14), on the other hand, differs. Different letters represent significant differences in percentage of mating among the different associations.

Males mounted females, pheromone silenced, sham-treated and non-manipulated nymphs equally often (Kruskal Wallis: χ^2 ^= 1.749, *P *= 0.626; Figure 1a). However, 92.9% of the females and 66.7% of the nymphs with blocked dorsal abdominal glands were mated and received sperm. No mating, or sperm transfer, was observed in the control and sham-treated nymph groups (Figure 1b). The percentage of mating with females (F) and silenced nymphs (SN) did not differ (Kruskal Wallis: χ^2 ^= 37.255, *P *< 0.001, followed by Mann Whitney U-test Z = -1.656, *P *= 0.274), but was significantly higher than when mating with nymphs, which were able to release alarm pheromone, that is control (*N*) and sham-treated nymphs (STN; Mann Whitney U-test: *Z*_N/STN _= 0, *P *= 1; *Z*_F/N _= -4.735, *P *< 0.001; *Z*_F/STN _= -4.837, *P *< 0.001; *Y*_SN/N _= -3.498, *P *< 0.004; *Y*_STN/SN _= -3.6, *P *< 0.003; Figure 1b).

### Single sensillum recordings

Stimulation with a nymphal bed bug head space extract elicited a significant response of ORNs housed in both grooved- and smooth-peg sensilla (25 and 58 Hz, respectively; Mann-Whitney: *Z*_Csensilla _= -2.556, *P *< 0.01 and *Z*_Dsensilla _= -3.098, *P *< 0.001). No response was observed in trichoid sensilla (Mann-Whitney: *Z*_Esensilla _= -1.023, *P *= 0.318; data not shown), and no sexual dimorphism was observed in the responses between males and females.

**Figure 2:**
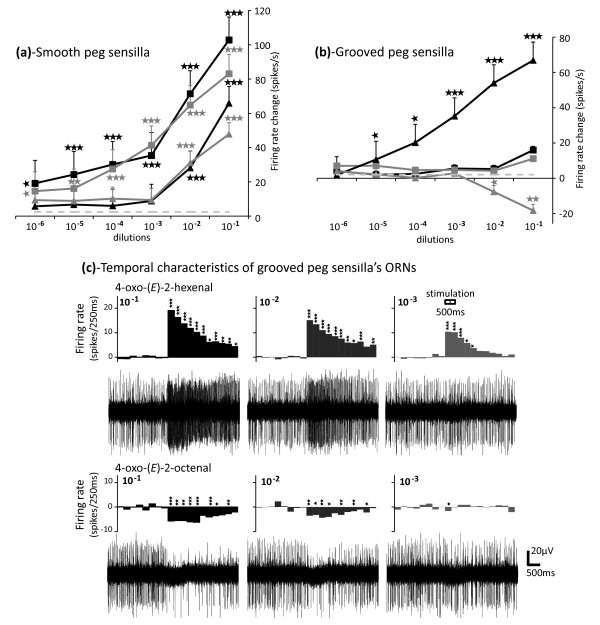
**Dose-dependent responses of olfactory receptor neurons housed in antennal olfactory peg sensilla of *Cimex lectularius***. (A, B) Extracellular recordings of olfactory receptor neurons housed in (a) smooth- and (b) grooved-peg sensilla reveal dose-dependent responses to (*E*)-2-hexenal (black squares), (*E*)-2-octenal (grey squares), 4-oxo-(*E*)-2-hexenal (black triangles) and 4-oxo-(*E*)-2-octenal (grey triangles). The points represent an average over six to 26 replicates and error bars correspond to the standard error of mean. (c) Temporal response characteristics of olfactory receptor neurons housed in the grooved peg sensilla. Histogram bars represent firing rate changes due to injection of 4-oxo-(*E*)-2-hexenal (*n *= 15) and 4-oxo-(*E*)-2-octenal (n = 6) within a sampling period of 250 ms each. The 500 ms stimulation started 2 s after the beginning of the recording (9th and 10th bins). Asterisks indicate statistically significant responses compared to the control, calculated by Mann Whitney U-test with * *P *< 0.05, ** *P *< 0.01 and *** *P *< 0.001.

The four nymph-emitted compounds, (*E*)-2-hexenal, (*E*)-2-octenal, 4-oxo-(*E*)-2-hexenal and 4-oxo-(*E*)-2-octenal, elicited dose-dependent responses in ORNs of smooth peg sensilla. However, only (*E*)-2-hexenal and (*E*)-2-octenal elicited significant responses at the lowest concentrations tested (10^-4 ^to 10^-6 ^dilution steps), compared to control (Figure 2a). Olfactory receptor neurons housed in grooved peg sensilla were specifically tuned to 4-oxo-(*E*)-2-hexenal and 4-oxo-(*E*)-2-octenal (Figure 2b and 2c). 4-Oxo-(*E*)-2-hexenal elicited a dose-dependent excitatory response, with significant responses observed at 10^-5 ^to 10^-1 ^concentrations compared to control. In contrast, 4-oxo-(*E*)-2-octenal elicited inhibitory dose-dependent responses, with significant responses observed only at 10^-2 ^and 10^-1 ^concentrations (Figure 2b and 2c). In light of these results, (*E*)-2-hexenal, (*E*)-2-octenal and 4-oxo-(*E*)-2-hexenal seem to be biologically relevant signals, and the following behavioural experiment was conducted using these compounds.

### Addition of alarm pheromone

**Figure 3:**
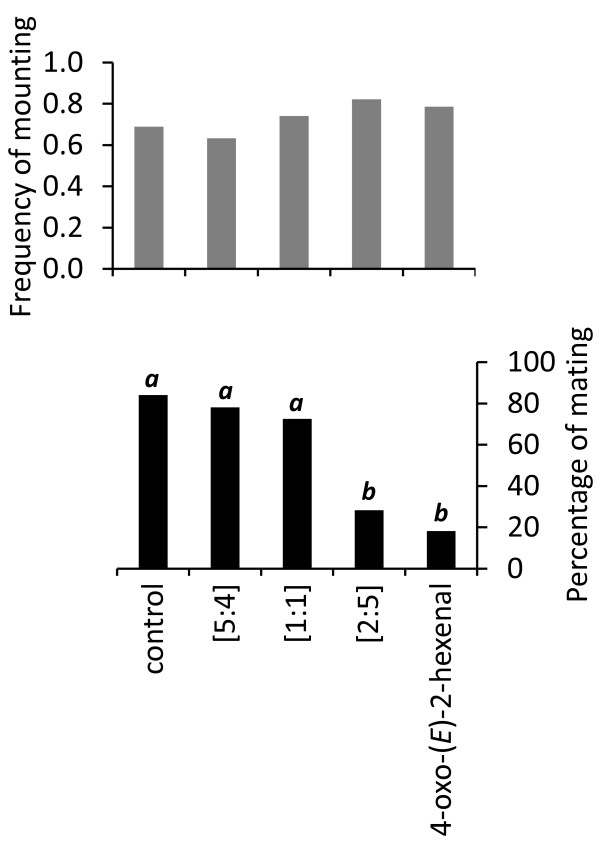
**Behavioural experiments on alarm pheromone effect when puffed just after the *Cimex lectularius *male mounted a female**. The male mating behaviour is regulated by nymph-emitted pheromone cues only. (a) Frequency of male mounting on females with covered methathoracic glands did not differ between the five experimental groups (*n *= 27-30; Kruskal-Wallis test: χ^2 ^= 5.322, *P *= 0.256) before puffing. (b) Stimulation with a male [5:4] (*n *= 19) or a female [1:1] (*n *= 20) ratio of (*E*)-2-hexenal:(*E*)-2-octenal did not significantly alter the percentage of male mating with these females, compared to control. However, stimulation with either the nymph-specific ratio of these aldehydes [2:5] (*n *= 23)) or 4-oxo-(*E*)-2-hexenal (*n *= 22) caused a significant decrease in percentage of mating (Kruskal-Wallis test: χ^2 ^= 35.602, *P *< 0.001; followed by Mann Whitney U-tests).

Transforming the females into silenced females by blocking their metathoracic glands did not affect male mounting behaviour (Kruskal Wallis: χ^2 ^= 5.322, *P *= 0.256; Figure 3a). However, stimulation with alarm pheromone onto a male/female pair induced different numbers of matings, depending on the compounds and ratios used (Kruskal Wallis: χ^2 ^= 35.602, *P *< 0.001; Figure 3b). A pair-wise comparison revealed that the male percentage of mating decreased significantly after stimulation with 4-oxo-(*E*)-2-hexenal (18.2%; Mann Whitney U-test: *Z *= -4.280, *P *< 0.001) and with (*E*)-hexenal: (*E*)-octenal at the nymph-specific ratio of 2:5 (28.3%; Mann Whitney U-test: *Z *= -3.664, *P *< 0.001; Figure 3b) compared to control (84.2%). In contrast, stimulation with (*E*)-hexenal: (*E*)-octenal at the female and male ratio of 5:4 and 1:1, respectively [Liedtke *et al*., unpublished data], did not affect the percentage of mating: 78.1% (Mann Whitney U-test: *Z *= -0.990, *P *= 0.411) and 72.5% (Mann Whitney U-test: *Z *= -1.222, *P *= 0.314), respectively, compared to control (84.2%).

## Discussion

This study demonstrates that the alarm pheromone produced by the dorsal abdominal glands of *C. lectularius *nymphs is detected by males and acts as a protection against traumatic extragenital insemination.

Male bed bugs mount any moving object of the size of a fed female bed bug [8] without discrimination of sex [5,10], developmental stage (this study) or even species [16] prior to physical contact [3,4]. These observations are corroborated by our study in which males mounted blood-fed females and nymphs with the same frequency (Figure 1a). However, when in physical contact, the male usually responds by a quick dismount when the mounted individual is a male [10] or a nymph (Figure 1b). By mating with a mistaken individual, the male wastes energy and sperm, and thereby decreases his reproductive success. The possible cost for the mounted individual may be mechanical damage such as gut perforation and the increased risk of infection by pathogens [6,17]. Females have physiological and anatomical adaptations, such as the spermalege, which reduces these fitness costs imposed during mating [4,17]. However, even for females, traumatic insemination carries a measurable fitness cost [7], which may be higher for males [10] and nymphs who lack such adaptations. It is therefore crucial for the nymphs to state their identity to males in order to avoid traumatic extragenital insemination. This statement is strengthened by our observation of silenced nymphs, which were unable to emit their alarm pheromone, and hence were mated (Figure 1b). To confirm the hypothesis that nymphs state their identity via their alarm pheromone, we tried to disrupt the mating of silenced females by adding alarm pheromone signals that are associated with nymph emission. We found that both (*E*)-2-hexenal:(*E*)-2-octenal in a nymph-specific ratio [2:5] and 4-oxo-(*E*)-2-hexenal, which all elicit ORN responses at biologically relevant concentrations, are sufficient to deter males from copulating with the manipulated female (Figure 3b). In contrast, the female and male specific ratio of the two aldehydes did not elicit dismounting (Figure 3b). This non-repulsive effect of the alarm pheromone emitted by adults may be linked, as mentioned by [2], to the dual interpretation of this blend in relation to the amount emitted. Indeed, males, in addition to releasing alarm pheromone while mounted [10], adopt specific behaviours of non-acceptance by bending their abdomen and shaking their body in order to escape piercing (personal observations).

## Conclusions

The unusual mating behaviour of the bed bug, with traumatic insemination, gives new insights to their chemical communication systems. Like males avoiding homosexual mistakes [10], nymphs also emit an alarm pheromone to avoid being chosen as partner. By further analysing the alarm pheromone ratios and content, we find that a specific ratio of aldehyde compounds, characteristic of nymphs, are sufficient to avoid mating, but we also conclude that 4-oxo-(*E*)-2-hexenal produced specifically by the nymphs is detected by specific sensilla on the male antennae and may contribute on its own to a repellent effect on males. This double effect may give a stronger protection to the nymphs against mating and piercing mistakes. The chemical communication system of the bed bugs is only just unfolding, and further analyses on longevity costs to nymphs as well as males who have been pierced is a high priority to fully understand the picture of traumatic insemination.

## Methods

### Insects

Bed bugs were reared in plastic jars (30 mm id) covered by nylon netting (0.5 mm) and placed in an incubator (KB8400 FL, Termaks, Bergen, Norway) under a 12 h:12 h light:dark cycle at 25°C and 70% humidity, as previously described [14]. Bed bugs were fed every 1-2 weeks on defibrinated chicken blood using an *in vitro *feeding system as described by [18]. After the blood meal, last instar nymphs were isolated individually until they moulted as adults, to ensure virginity of the experimental individuals.

For single sensillum recordings, one-to-three weeks old unmated and unfed adults were used in order to pinpoint possible sexual dimorphism in ORN responses. For behavioural experiments, large nymphs and unmated females were fed on chicken blood for 10 min prior to each observation, whereas males had been unfed for 1-2 weeks.

### Single sensillum recordings

For single sensillum recordings, a bed bug was dorsally restrained under a Nikon Eclipse (E600-FN) microscope, which allowed us to identify individual sensilla at high magnification (×750), in order to test all the different functional types previously characterized by [14]. Using a piezoelectric micromanipulator (DC-3K, Märzhauser Wetzlar, Berlin, Germany), an electrolytically sharpened tungsten microelectrode was introduced into the shaft or base of a sensillum and the reference tungsten electrode was inserted into the head capsule through the neck region. The recording electrode was connected to a preamplifier (×10, Syntech, Kirchzarten, Germany) and the electrical signals were fed through an analogue-digital signal converter (IDAC-4, Syntech) and then visualized and recorded on a computer using the Autospike (v3.3, Syntech, Germany) software.

The insect antenna was placed in a humidified charcoal-filtered air stream delivered at 1 m/s via a glass tube (6 mm id), and a stimulus controller (CS-02, Syntech) permitted us to divert a 2 mL/s airflow through a Pasteur pipette, containing a stimulus, for 500 ms. The interval between each stimulation lasted until the neuronal activity reached the prestimulus frequency level.

The headspace collection of four large nymphs (Leidtke *et al. *unpublished data) was puffed over the antenna in order to measure the detection of the nymph-emitted volatiles. As the nymph head space collections were prepared in hexane, this solvent was used as a control during these experiments. Synthetic chemicals, (*E*)-2-hexenal (98%, Aldrich), (*E*)-2-octenal (>94%, Sigma), 4-oxo-(*E*)-2-hexenal and 4-oxo-(*E*)-2-octenal (96% and 98%, respectively, synthesized and supplied by E Hedenström, Mid Sweden University), were dissolved in paraffin oil (Merck, Darmstadt, Germany). During dose-response experiments, all compounds were tested in a series from the lower to the higher dilution and paraffin oil was used as a control.

### Behavioural experiments

#### Male mounting of nymphs

In order to test differences in male attraction to females and nymphs, a recently fed female or late instar nymph was carefully placed into the centre of an arena (glass Petri dish, 50 mm diameter), with filter paper (renewed after each experiment) covering the base to facilitate the bed bugs' movement. A randomly selected unfed virgin male was then introduced and the behaviour of the couple was monitored for 5 min. The male mounting and dismounting behaviours were observed and timed to one tenth of a second, using a customized data logger application written in Delphi 6.0. All mounted individuals were inspected at the end of the experiment for the presence of ejaculate in the abdomen as a proof of a mating resulting from a successful mounting. All the behavioural experiments were carried out at room temperature in dimmed light conditions.

#### Removal of nymph pheromone

When testing the effect of nymph odour on male mounting behaviour, large nymphs were randomly divided into three treatment groups: silenced nymphs, sham nymphs and control. The silenced nymphs' dorsal abdomen was carefully coated with nail polish in order to cover their dorsal abdominal glands, as described in [10], whereas sham nymphs had the sides of their abdomen painted. The last group of nymphs, as well as a group of females, were not manipulated. Observations were conducted as described above and individuals whose natural behaviour was altered by the painting were discarded.

#### Addition of alarm pheromone

Prior to the observation, the methathoracic glands of an unfed female were covered by nail polish to prevent emission of alarm pheromones. In order to mimic a male/nymph interaction, odours were mechanically puffed onto a male/female pair as soon as the male mounted the silenced female. The couple was exposed to a single puff of stimulus delivered via a Pasteur pipette equipped with a 5 mL rubber bulb as a manual release system. The Pasteur pipette contained a filter paper strip (~5 × 15 mm) impregnated with 10 μL of a stimulus solution. The chemicals used in the behavioural experiments were diluted singly in hexane (99%), down to a 100 ng/μL level (10^-4 ^dilution step). The (*E*)-2-hexenal: (*E*)-2-octenal blend was then formulated to obtain three different ratios: a female ratio of 5:4, a male ratio of 1:1 and a nymph ratio of 2:5 [Liedtke *et al*., unpublished data]. The three ratios of aldehydes, the oxo-aldehyde and a hexane control were tested on four different groups of male/female pairs, and observations were conducted in the same manner as described above.

### Analysis

#### Single sensillum recordings

The antennal olfactory system of bed bugs is composed of nine grooved peg sensilla, six smooth peg sensilla and 29 trichoid sensilla [19]. As bed bug sensilla contain a high number of ORNs, we were unable to differentiate single ORNs using the shape and amplitude of their spikes (action potentials) (Figure 2c) [14]. For this reason, the total number of all spikes was manually counted for each recording, allowing us to assess the overall ORN activity elicited by stimulation within a specific sensillum. The net number of spikes per second (number of spikes 500 ms before stimulus onset subtracted from the number of spikes 500 ms after stimulus onset and then multiplied by two) in response to the blank were subtracted from the net number of spikes in response to stimuli. In addition, the mean temporal pattern of response was assessed by counting the total number of spikes within 250 ms duration bins. For this, the mean spike frequency of the eight first bins before stimulation (2 s) was calculated and was subtracted from each bin in order to obtain the net firing rate change per bin.

For response analysis, the Mann Whitney U-test was used to compare the response to test stimuli and solvent (Figure 2a and 2b). The same test was used for temporal response analysis, in which firing rate changes to the test stimulus were compared bin by bin (250 ms interval) to firing rate changes to the control stimulus (Figure 2c). All statistical analysis was performed with SPSS software (release 17.0, SPSS, USA).

#### Behavioural experiments

The number of trials with mountings were added and divided by the total number of trials to give the mounting frequency. The percentage of successful mountings (matings) was calculated by dividing the number of mated partners by the number of mounted partners.

The Kruskal Wallis test was applied to compare general differences in sample groups larger than two. It was followed by the Mann Whitney U-test in order to statistically compare the proportions between two samples. The time of mounting was log transformed in order to reach normality (Kolmogorov Smirnov test) and as the variance was statistically similar between the two groups (Levene test), one way analysis of variance was then applied. All the statistical analyses were performed with SPSS software (release 17.0, SPSS, USA).

## Abbreviations

ORN: olfactory receptor neuron; F: females; SN: silenced nymphs; N: controls; STN: sham-treated nymphs.

## Authors' contributions

VH participated in the design of the study, carried out the electrophysiological study, contributed in the behavioural studies, performed the data and statistical analysis and drafted the manuscript. CR conceived the behavioural design of this study, helped in the data interpretation and drafted the manuscript. RI helped in the data interpretation and edited the manuscript. All authors read and approved the final manuscript.
